# 

**Published:** 1890-06

**Authors:** 


### TABLE OF CONTENTS.

ORIGINAL COMMUNICATIONS. PAGE

Reflex Ocular and Facial Symptoms of Nasal Disease. By EDWARD S. PECK, A.M., M.D., Visiting Surgeon to Charity Hospital; Consulting Surgeon to Bellevue Hospital, Out-door Department; Ophthalmic Surgeon to the Montefiore Home for Chronic Invalids; Member of the New York Pathological Society; Fellow of the New York Academy of Medicine, etc. . . . . 321

A New Method for the Treatment of Fractures of the Maxillæ. By EDWARD H. ANGLE, D.D.S., Minneapolis . . . . . 330

Oral Surgery Clinic.—Mercy Hospital, Chicago. Service of PROFESSOR JOHN S. MARSHALL . . . . . 337

Dental Education. By C. STODDARD SMITH, D.D.S. . . . . . 339

REPORTS OF SOCIETY MEETINGS.

Chicago Dental Club . . . . . 348

New York Odontological Society . . . . . 362

EDITORIAL.

Society Meetings . . . . . 378

FOREIGN CORRESPONDENCE . . . . . 380

DOMESTIC CORRESPONDENCE . . . . . 383

CURRENT NEWS . . . . . 384

